# Olaquindox-Induced Liver Damage Involved the Crosstalk of Oxidative Stress and p53 In Vivo and In Vitro

**DOI:** 10.1155/2020/8835207

**Published:** 2020-12-18

**Authors:** Daowen Li, Xingyao Pei, Xiaoling Qin, Xinyu Liu, Cun Li, Liuan Li, Chongshan Dai, Xilong Xiao, Shusheng Tang

**Affiliations:** ^1^Department of Pharmacology and Toxicology, College of Veterinary Medicine, China Agricultural University, Yuanmingyuan West Road No. 2, Haidian District, Beijing 100193, China; ^2^Tianjin Key Laboratory of Agricultural Animal Breeding and Healthy Husbandry, College of Animal Science and Veterinary Medicine, Tianjin Agricultural University, Jinjing Road No. 22, Xiqing District, Tianjin 300384, China

## Abstract

Olaquindox (OLA), a member of the quinoxaline-N,N-dioxide family, has been widely used as a growth-promoting feed additive and treatment for bacterial infections. The toxicity has been a major concern, and the precise molecular mechanism remains poorly understood. The present study was aimed at investigating the roles of oxidative stress and p53 in OLA-caused liver damage. In a mouse model, OLA administration could markedly cause liver injury as well as the induction of oxidative stress and activation of p53. Antioxidant N-acetylcysteine (NAC) inhibited OLA-induced oxidative stress and p53 activation in vivo. Furthermore, knockout of the p53 gene could significantly inhibit OLA-induced liver damage by inhibiting oxidative stress and the mitochondria apoptotic pathway, compared to the p53 wild-type liver tissue. The cell model in vitro further demonstrated that p53 knockout or knockdown in the HCT116 cell and L02 cell significantly inhibited cell apoptosis and increased cell viability, presented by suppressing ROS production, oxidative stress, and the Nrf2/HO-1 pathway. Moreover, loss of p53 decreased OLA-induced mitochondrial dysfunction and caspase activations, with the evidence of inhibited activation of phosphorylation- (p-) p38 and p-JNK and upregulated cell autophagy via activation of the LC3 and Beclin1 pathway in HCT116 and L02 cells. Taken together, our findings provided a support that p53 primarily played a proapoptotic role in OLA-induced liver damage against oxidative stress and mitochondrial dysfunction, which were largely dependent on suppression of the JNK/p38 pathway and upregulation of the autophagy pathway via activation of LC3 and Beclin1.

## 1. Introduction

Olaquindox (OLA) is one member of the quinoxaline 1,4-dioxide (QdNOs) family. A series of QdNOs compounds have been identified and widely used in agricultural and medicinal fields. OLA contains many pharmacological activities, such as antimicrobial, antitumoral, antitrypanosome, anti-inflammatory, and antioxidant activities [1, 2]. OLA has been widely used as one of the growth-promoting feed additives and treatment for bacterial infections in the animal clinic. The antibacterial mechanism of OLA is to inhibit DNA synthesis, which is particularly effective against Gram-negative bacteria (E. coli, Pasteurella, Shigella, etc.). It is also sensitive to some Gram-positive bacteria such as Staphylococcus and Streptococcus. The growth-promoting mechanism of OLA is mainly through strengthening the activity of digestive enzymes in animals, which can promote protein assimilation and increase feed conversion rate. However, the potential toxicity of OLA, such as genotoxicity, cytotoxicity, reproduction toxicity, nephrotoxicity, and hepatotoxicity, has seen wide concern [3]. OLA has been widely used in animal feed and aquatic environments, which could pollute animal food and water sources. Furthermore, high concentrations of OLA in manure applied to agriculture could eventually lead to their residues in surface water, which will further damage animal and human health.

Previous studies have demonstrated that OLA-induced cytotoxicity and genotoxicity were dependent on the production of reactive oxygen species (ROS) and DNA damage [4]. It has also been reported that OLA induced cytotoxicity and tissue damage involving apoptosis and autophagy [5, 6]. There are multiple evidences to demonstrate that the excessive production of ROS is a major contributor in QdNOs-induced oxidative stress and DNA damage, such as OLA, quinocetone, carbadox, 2-benzoyl-3-phenyl-6,7-dichloroquinoxaline 1,4-dioxide (DCQ), and mequindox [7–10]. It was later found to be due to the special structure of the QdNOs molecule's N-O group [11, 12]. In addition, the pathway of caspase-dependent mitochondria has been suggested to contribute to the cell apoptosis induced by OLA and quinocetone [13]. OLA could activate cell autophagy which was dependent on the activation of the ROS/JNK pathway [14]. Moreover, OLA played an important role in cell cycle arrest, which involved the activation of p53, GADD45a, p21, cyclin A, and Cdk2 protein [15, 16]. Our previous study found that p21 knockdown regulated OLA-induced mitochondrial apoptosis and cell cycle arrest through the upregulation of AKT and downregulation of Nrf2/HO-1 pathways [17].

p53, a tumor suppressor, could be activated by a variety of stress stimuli, including oxidative stress, which orchestrates an extremely complex transcriptional program that inhibits proliferation and promotes apoptosis [18]. It has been demonstrated that p53 played an important role in quinocetone-induced apoptosis [19]. However, El-Khatib et al. showed that DCQ-induced cell apoptosis was independent on the activation of p53 and p21 [20]. Yang et al. suggested that OLA-induced apoptosis was involved in the activation of p53 in human 293 cells [15, 21]. However, the precise molecular mechanism remains unknown. The present study is aimed at investigating the crosstalk of oxidative stress and p53 in OLA-induced liver damage and excavating the underlying molecular mechanisms in vitro and in vivo.

## 2. Materials and Methods

### 2.1. Chemicals and Reagents

The OLA (purity ≥ 98%) was provided by the China Institute of Veterinary Drug Control (Beijing, China). 3-(4,5-Dimetylthiazol-2-yl)-2,5-diphenyltetrazolium bromide (MTT) and N-acetylcysteine (NAC) were purchased from Sigma-Aldrich (St. Louis, MO, USA). Dulbecco's modified Eagle's medium (DMEM) and fetal bovine serum (FBS) were purchased from Life Technologies Corporation (Grand Island, NY, USA). Rhodamine (Rh) 123 and dichlorofluorescein diacetate (DCFH-DA) were offered by Beyotime Institute of Biotechnology Co., Ltd. (Haimen, China). All other chemicals and reagents were of the highest analytical grade.

### 2.2. Animal Experiments

All animal experiments were approved by the Institutional Animal Ethics and Use Committee at the China Agricultural University. Male adult C57BL/6 mice (20 to 22 g) were obtained from Vital River Animal Technology Co., Ltd. (Beijing, China). p53 knockout and paired wild-type mice (20-22 g) were purchased from Beijing Biocytogen Co., Ltd. (Beijing, China). The mice were housed under standard laboratory conditions and allowed free access to water and food during all experimental periods. Mice were randomly divided into control, OLA100, OLA200, OLA400, NAC, NAC+OLA (*n* = 10 in each group). OLA was suspended in 0.5% carboxyl methyl cellulose sodium. OLA groups were orally administrated with OLA at the doses of 100, 200, and 400 mg/kg/day, respectively, for 28 consecutive days, while the NAC+OLA group was gavaged with NAC (100 mg/kg) 2 h in advance; followed by OLA (400 mg/kg) administration, the control group was orally administered as the equal vehicle, whereas the p53 knockout (p53-/-) and paired wild-type (p53 wt) mice were administrated with OLA at the concentration of 400 mg/kg/day for 28 consecutive days or the equal vehicle. After 24 h following the last gavage, all mice were euthanized by intraperitoneally injecting a high dose of sodium pentobarbital (80 mg/kg) (Sigma-Aldrich, St. Louis, MO, USA). The blood samples were collected, and the serum was separated by centrifugation (3000 g for 15 min) and stored at −80°C until assayed.

### 2.3. Measurement of Serum Enzymes Aspartate Transaminase (AST) and Alanine Aminotransferase (ALT) and Biomarkers of Oxidative Stress and the Activity of Caspase 3/9

Levels of serum aspartate transaminase (AST) and alanine aminotransferase (ALT) were detected using commercially standard diagnostic kits (Nanjing Jiancheng Bio-Corporation) via an Automated Chemical Analyzer (Hitachi 7080, Hitachi High-Technologies Corporation). The superoxide dismutase (SOD), catalase (CAT) activity, and malondialdehyde (MDA) level were measured using specific assay kits as per the respective manufacturer's instructions (Nanjing Jiancheng Bio-Corporation). The activities of caspase 3/9 in the liver tissues were determined using the caspase 3/9 activity detection ELISAPlus kit (Roche Applied Sciences, Basel, Switzerland).

### 2.4. Histological Analysis

The liver of each mouse was fixed in formalin for more than 48 h at room temperature. Then, the fixed liver tissues were washed with tap water, dehydrated with gradient alcohol, paraffin-embedded, and partially stained with hematoxylin-eosin (HE) according to our previous study [22]. Another portion was stored at 4°C for immunohistochemical staining. For the immunohistochemical analysis, the liver tissue slices were deparaffinized in a thermotank at 60°C for 30 min and dehydrated in graded alcohol. The antigens were repaired by microwave, soaked in 3% H_2_O_2_ for 10 min, and blocked with 5% goat serum for 30 min. The primary antibody was directly incubated overnight. The primary antibody concentrations were as follows: rabbit polyclonal anti-Bax (1 : 200) and Bcl-2 (1 : 100). The next day, the samples were incubated with biotinylated goat anti-rabbit IgG-horseradish peroxidase (HRP) conjugated for 30 min. Afterwards, the sections were developed with DAB for 3 min, followed by hematoxylin staining for 5 min, washed twice, underwent alcohol gradient dehydration, and mounted in a xylene-based mountant. Immunohistochemical results were evaluated by the software of ImageJ 1.46 (National Institute of Mental Health, Bethesda, MD, USA).

### 2.5. Transmission Electron Microscopy Examination

The ultrastructure of the liver was examined as our previous study described [23]. In short, the liver tissue was cut into 1 mm^3^ tissue pieces and placed in a 2.5% glutaraldehyde solution for 2 h, followed by fixing with 1% osmium tetroxide for 1.5 h. Then, the tissue was dehydrated with alcohol, transferred to a 90% ethanol : 90% acetone (1 : 1) mixed solution for 15 min, and placed in 100% acetone for 10 min. Subsequently, the tissues were treated with 100% acetone and embedding agent (Epon812) for 2 h. The next day, the tissue was embedded with Epon812. After drying in an oven at 37°C, ultrathin sectioning was performed. The section thickness of 50-70 nm was stained with 4% uranyl acetate and lead citrate for 15 min. The ultrastructure was observed under a transmission electron microscope (JEOL Ltd., Tokyo, Japan).

### 2.6. Cell Culture and Treatments

Human HL-7702 (L02) cell line was purchased from the Shanghai Institute of Cell Biology, Chinese Academy of Sciences. HCT116 (p53+/+) and HCT116 (p53-/-) cell lines were provided by Professor Jun Tang, China Agricultural University. Cells were incubated in RPMI-1640 (Gibco, Grand Island, NY, USA) complemented with 10% fetal bovine serum and 100 U/mL penicillin and streptomycin in a cell incubator at 37°C with 5% CO_2_. OLA was prepared as previous study described [4]. The cells were treated with OLA for 24 h.

### 2.7. Cell Viability Assay

Cell viability was assessed via MTT assay according to the manufacturer instructions. In short, cells (1 × 10^4^) were seeded into 96-well culture plates for 24 h and treated with different concentrations of OLA. After 24 h, the OLA was removed, and cells were incubated with 100 *μ*L fresh RPMI-1640 containing 500 *μ*g/mL MTT for 4 h. Then, 100 *μ*L DMSO was added to dissolve the formazan crystals. The formation of formazan was recorded by a microplate reader at 570 nm (Molecular Devices, Sunnyvale, CA, USA).

### 2.8. Plasmid Transfection

The p53 interfering plasmid was preserved in our laboratory: p53 interference target sequence (shRNA p53)—GACTCCAGTGGTAATCTAC; p53 negative control plasmid sequence (vector)—ACTACTGGACTCTTGGCA. L02 cells (1 × 10^5^) were cultured in six-well plates for 24 h and mixed with 2 *μ*g plasmid (shRNA or vector) and 6 *μ*L of transfection reagent (Roche, Basel, Switzerland), according to the manufacturer's instructions. After 48 h, the cells were collected for western blot or other experiments.

### 2.9. Measurement of Apoptosis

Annexin V-FITC/PI staining: apoptosis was detected using Annexin V-FITC/PI detection kit (Vazyme Biotech Co., Ltd., Nanjing, China). After treatment with OLA, cells were digested by 0.25% trypsin without EDTA. Cells were resuspended in Binding Buffer and Annexin V-FITC/PI staining was added. Apoptosis cells were measured by flow cytometer (BD FACSAria™). The data were analyzed using BD FACSDiva software (BD Biosciences, San Jose, CA, USA). Hoechst 33342 staining: cells treated with OLA for 24 h. After staining in the dark with 1 *μ*g/mL Hoechst 33342 (Vigorous Biotechnology, Beijing, China) for 20 min, the cells were observed under a fluorescence microscope (Leica Microsystems, Wetzlar, Germany). Cells which showed chromatin condensation were counted as apoptotic cells.

### 2.10. Reactive Oxygen Species (ROS) Measurement

ROS level was determined by the fluorescence probe DCFH-DA. After OLA treatment, the cell was incubated with KRBBS buffer containing 10 *μ*g/mL DCFH-DA staining for 20 min. Cells were washed with PBS for three times and observed with a fluorescence microscope (Leica Microsystems, Wetzlar, Germany).

### 2.11. Measurement of Mitochondrial Membrane Potential (MMP)

Rhodamine 123 (Rh123) is a green fluorescent dye that could be used as an indicator of MMP (Sigma-Aldrich, St. Louis, MO, USA). After OLA treatment, cells were stained with Rh123 (10 *μ*g/mL) for 30 min in the dark. Subsequently, cells were washed with PBS for three times and observed under a fluorescence microscope (Leica Microsystems, Wetzlar, Germany). The images were evaluated using the software of ImageJ (Media Cybernetics, Inc., Silver Spring, MD).

### 2.12. Western Blotting Assay

Protein extraction from cells and tissues and western blot are according to our previous description. The concentrations of protein were measured with a BCA protein assay kit (Beyotime Institute of Biotechnology Co., Ltd, Haimen, China). The concentrations of primary antibodies were prepared as follows: rabbit polyclonal antibodies against Beclin1 (1 : 1000), p53 (1 : 1000; Santa Cruz, CA, USA), Bax (1 : 2000), Bcl-2 (1 : 1000), LC3 (1 : 1000) (ProteinTech Group, Inc., Chicago, IL, USA), caspase 3 (1 : 500), caspase 9 (1 : 500; Cell Signaling Technology, Beverly, MA, USA), and PARP (1 : 1000; Beyotime, Haimen, China) and mouse polyclonal antibodies against *β*-actin (1 : 1000) and Cytochrome C (Cyt C) (1 : 1000, Zhongshan Golden Bridge, Beijing, China). The bands were detected by ECL luminescent (Vigorous Biotechnology, Beijing, China) and analyzed using the software of ImageJ (National Institute of Mental Health, Bethesda, MD, USA).

### 2.13. Monodansylcadaverine (MDC) Measurement

MDC is a fluorescent dye used as a tracer for the autophagocytic vesicle agent. In brief, cells were inoculated in 6-well plates with 1 × 10^6^ cells per well. After OLA treatment for 24 h, MDC (final concentration: 50 *μ*M) was added to each well and incubated in a constant temperature incubator at 37°C 20 min in darkness. The result was observed with a fluorescence microscope (Leica Microsystems, Wetzlar, Germany).

### 2.14. Statistical Analysis

All data were presented as mean ± SD. Figures were performed via GraphPad Prism 8.0 (GraphPad Software, Inc., La Jolla, CA, USA). The software of SPSS (SPSS 17.0, Inc., Chicago, USA) was used for statistical analysis. A *p* value less than 0.05 (*p* < 0.05) was considered as significant.

## 3. Results

### 3.1. OLA Induced Hepatic Dysfunction, Oxidative Stress, and p53 Activation in Mice

**Figure 1:**
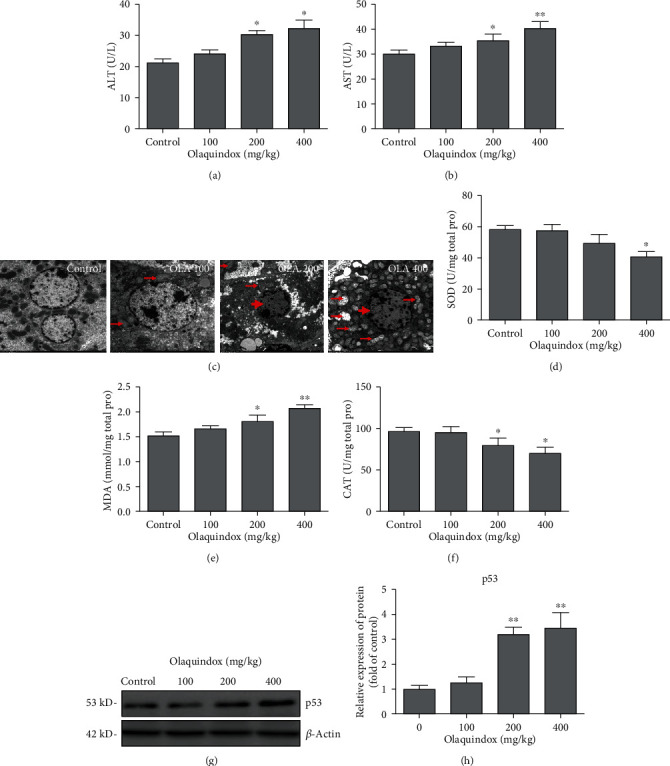
OLA induced hepatic dysfunction, oxidative stress, and p53 activation in mice. C57BL/6 mice were orally administrated with OLA at the doses of 100, 200, and 400 mg/kg for 28 consecutive days. (a) ALT serum levels. (b) AST serum levels. (c) Effect of OLA on ultrastructure changes in mouse liver observed by electron microscope (1200x). (d–f) Impact of OLA treatment on SOD activity, MDA levels, and CAT activity. (g) p53 protein expressions in the liver tissue of mice were analyzed by western blotting. (h) The densitometry analysis of p53 by ImageJ 1.46 software. The results represent mean ± SD (*n* = 10). Compared with the control, ^∗^*p* < 0.05, ^∗∗^*p* < 0.01.

Mice gavaged with OLA at doses of 200 and 400 mg/kg/day for 28 days significantly increased the activities of AST and ALT (Figures 1(a) and 1(b)), respectively. Transmission electron microscopy results of mouse liver tissue showed that normal hepatocytes in the control group were in the middle, with abundant mitochondria and clear cell boundaries. With the increase of the OLA dose, the cytoplasm was dissolved followed with mitochondrial swelling (small arrow) and chromatin consolidation (big arrow) (Figure 1(c)). Oxidative stress biomarkers of SOD, CAT, and MDA were assessed in OLA-induced liver damage. Compared with the control, in the OLA 200 mg/kg and 400 mg/kg groups, the MDA level increased from 1.55 ± 0.04 mmol/mg to 1.72 ± 0.13 mmol/mg and 1.92 ± 0.05 mmol/mg (Figure 1(e)). The activity of SOD in the OLA 400 mg/kg group decreased from 59.2 ± 3.2 U/mg to 43.2 ± 4.2 U/mg (Figure 1(d)). The activity of CAT decreased from 101.3 ± 2.2 U/mg to 81.7 ± 5.6 U/mg and 71.3 ± 5.4 U/mg in OLA 200 mg/kg and 400 mg/kg groups (Figure 1(f)). Furthermore, the results showed that OLA activated p53 in a dose-dependent way (Figure 1(g)).

### 3.2. Inhibition of Oxidative Stress Attenuated p53 Activation Induced by OLA

**Figure 2:**
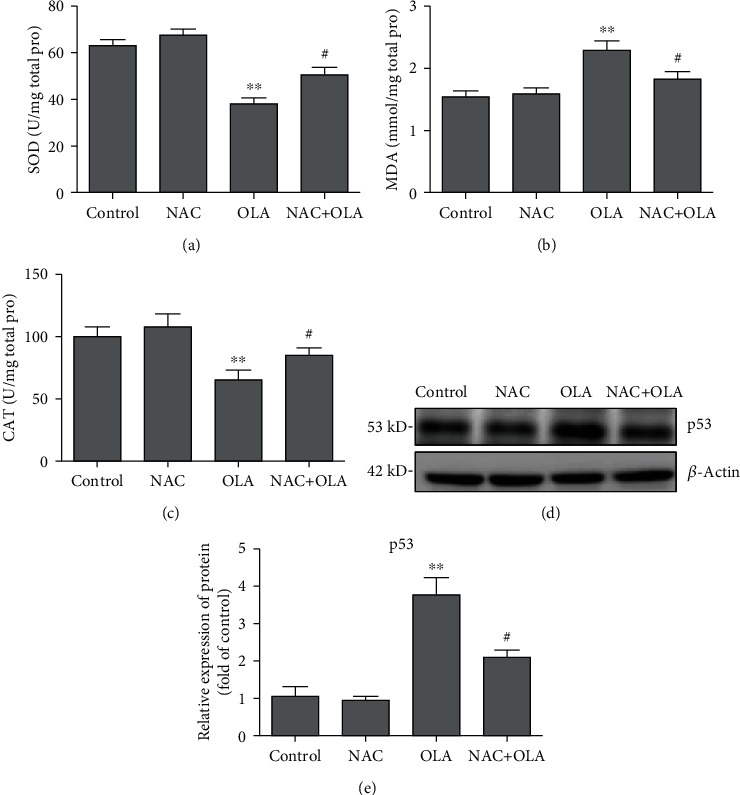
Inhibition of oxidative stress attenuated p53 activation induced by OLA. (a–c) The activities of SOD and CAT and the level of MDA in NAC pretreatment OLA-induced mice. (d, e) p53 protein expression in the liver tissue of mice was analyzed by western blotting from the control and NAC pretreated OLA groups. The results represent mean ± SD (*n* = 10). Compared with the control, ^∗∗^*p* < 0.01; compared with the OLA alone group, ^#^*p* < 0.05.

**Figure 3:**
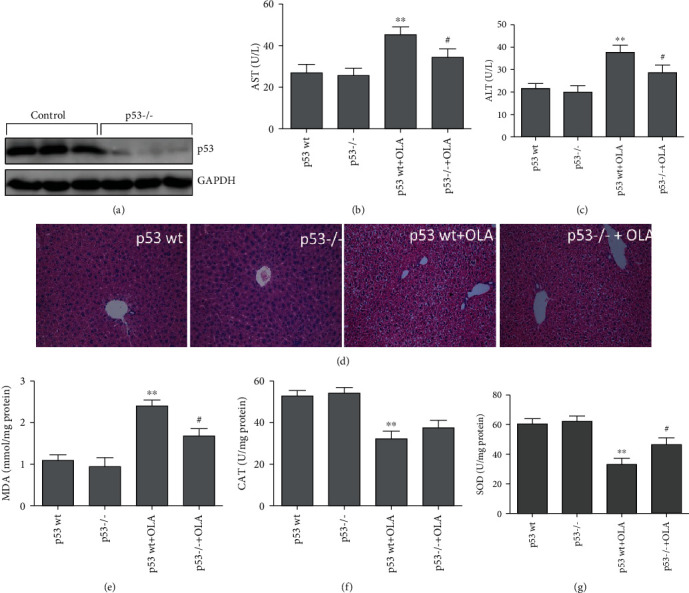
p53 knockout decreased OLA-induced liver damage and oxidative stress in mice. (a) p53 protein expression was analyzed by western blotting in p53 wild type and p53 knockout groups. (b, c) Effect of p53 on OLA-induced ALT and AST serum levels. (d) Histopathological images of HE-stained liver sections from OLA-treated p53 wild type and p53 knockout mice. (e–g) Effect of p53 on OLA-induced MDA levels and CAT and SOD activities in mice. The results represent mean ± SD (*n* = 10). Compared with the p53 wt group, ^∗∗^*p* < 0.01; compared with the p53 wt+OLA group, ^#^*p* < 0.05.

As shown in Figure 2, the result showed that in the absence of p53, the activities of SOD and CAT increased (Figure 2), compared to those of the OLA-alone group. Meanwhile, NAC pretreatment markedly attenuated OLA-induced hepatic p53 expression (Figure 3(d)).

### 3.3. p53 Knockout Decreased OLA-Induced Liver Damage and Oxidative Stress in Mice

p53 knockout mice were selected to demonstrate the role of p53 in OLA-induced damage. As shown in Figure 3(a), the results showed that the p53 protein was not detected in p53 knockout mice. OLA-induced serum ALT and AST levels of mice were significantly decreased in the p53-/- group, compared with the p53 wt+OLA group (Figures 3(b) and 3(c)). Histopathological results showed that the hepatic cords were arranged neatly, and the structure of the hepatocytes was intact and clear in OLA-untreated groups. However, p53 knockout attenuated OLA-induced liver tissue damage with the evidences of decrease in the disorder of hepatocyte arrangement, cytoplasmic lysis, and cytoplasmic vacuolization, compared with the p53 wt+OLA group (Figure 3(d)). As shown in Figure 3, p53 knockout effectively reduced the MDA level induced by OLA (Figure 3(e)), significantly increased SOD activity in liver tissues (Figure 3(g)), and showed no significant change in CAT activity (Figure 3(f)).

### 3.4. Depletion of p53 Attenuated OLA-Induced Mitochondrial Apoptosis Pathway

**Figure 4:**
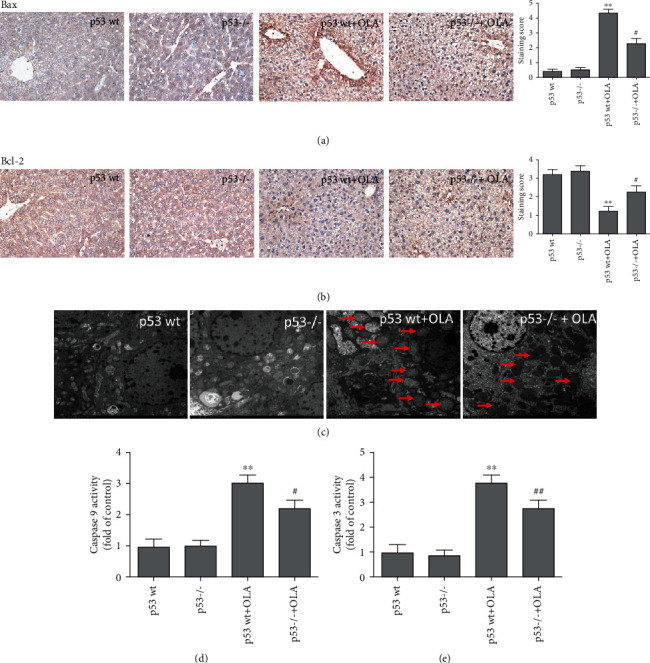
Depletion of p53 attenuated OLA-induced mitochondrial apoptosis pathway. (a, b) The expression of BAX and Bcl-2 was analyzed via immunohistochemistry. (c) Effects of p53 on OLA-induced ultrastructure changes in mouse liver observed by electron microscope (1200x). (d, e) Effects of p53 on OLA-induced activities of caspase 9 and caspase 3. The results represent mean ± SD (*n* = 10). Compared with the p53 wt group, ^∗∗^*p* < 0.01; compared with the p53 wt+OLA group, ^#^*p* < 0.05 and ^##^*p* < 0.01.

As shown in Figure 4, the expressions of Bax and Bcl-2 in the liver tissues were measured by immunohistochemical analysis. In mice treated with OLA (400 mg/kg) for 28 days, the expression of Bax was significantly increased, while the expression of Bcl-2 significantly decreased, compared with that of the p53 wt group (Figures 4(a) and 4(b)). However, p53 knockout decreased the OLA-induced expression of Bax and increased the expression of Bcl-2, compared with the p53 wt+OLA group (Figures 2(f) and 2(g)). Moreover, depletion of p53 attenuated OLA-induced mitochondrial swelling (red arrow) (Figure 4(c)). Meanwhile, the activities of caspase 9 and caspase 3 significantly decreased in the homogenates of OLA-treated liver tissue, compared with the p53 wt+OLA group (Figures 4(d) and 4(e)).

### 3.5. p53 Knockout or Knockdown Attenuated OLA-Induced Apoptosis in HCT116 and L02 Cells

**Figure 5:**
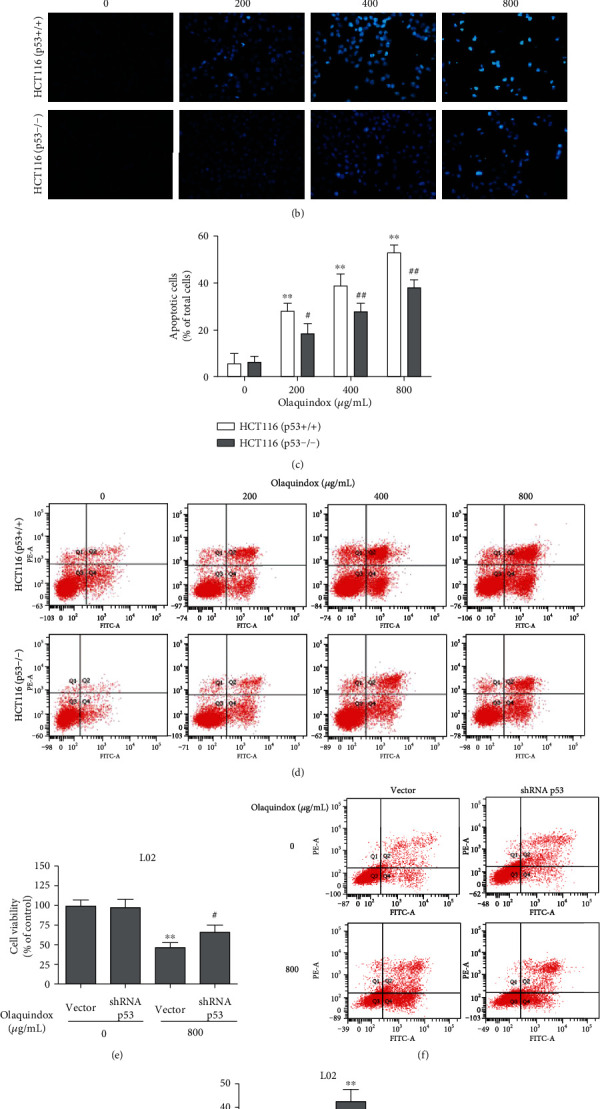
p53 knockout or knockdown attenuated OLA-induced apoptosis in HCT116 and L02 cells. (a) Cell viability of HCT116 cells was estimated by MTT assays. (b) HCT116 cells were stained with Hoechst 33342 and observed under an inverted fluorescence microscopy (400x). (c) The histogram depicted the mean apoptotic rate of HCT116 cells. (d) HCT116 cells were stained with Annexin V-FITC/PI and detected by flow cytometry. (e) Cell viability of L02 cells was estimated by MTT assays. (f) L02 cells were stained with Annexin V-FITC/PI and detected by flow cytometry. (g) The histogram depicted the mean apoptotic rate of L02. The results represent the means ± SD of three independent experiments. ^∗^*p* < 0.05 and ^∗∗^*p* < 0.01, compared with the control; ^#^*p* < 0.05 and ^##^*p* < 0.01, compared with the p53 normal group.

To further investigate the effect of p53 on OLA-induced cell death in vitro, we selected p53 deletion cell lines HCT116 (p53+/+) and HCT116 (p53-/-). As shown in Figure 5(a), the cell viability was significantly increased after p53 deletion, compared with the HCT116 (p53+/+) group. Hoechst 33342 staining results showed that the blue fluorescence and nuclear condensation in the p53 deletion group was markedly lower than that in the p53 normal group (Figure 5(b)). Compared with the HCT116 (p53+/+) group, the apoptosis rate decreased from 51.35 ± 4.28% to 38.74 ± 4.65% treated with 800 *μ*g/mL OLA in the p53 deletion group (Figures 5(d) and 5(e)). Therefore, we used the p53 interfering plasmid to interfere with the expression of p53 in L02 cells for verification. The results showed that interfering with p53 increased the cell viability induced by OLA and decreased the OLA-induced apoptosis in L02 cells (Figures 5(e)–5(g)), which were consistent with the results of HCT116 cells.

### 3.6. Loss of p53 Inhibited OLA-Induced Oxidative Stress In Vitro

**Figure 6:**
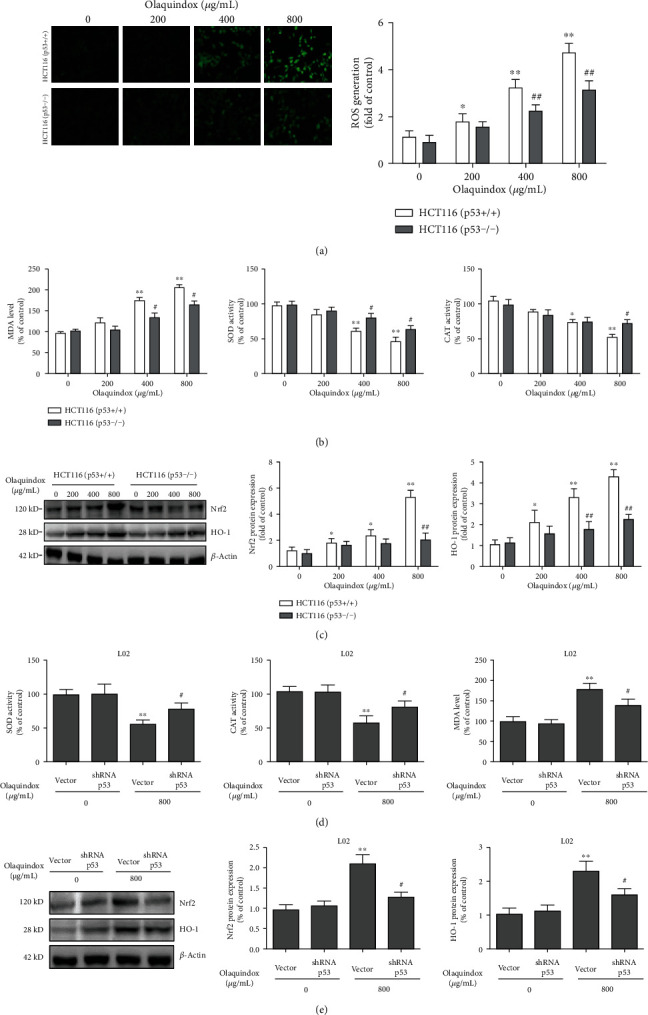
Loss of p53 inhibited OLA-induced oxidative stress in vitro. (a) HCT116 cells were incubated with 10 mM DCFH-DA for 30 min. The fluorescence intensity was observed by fluorescence microscope (400x). Fluorescence intensity was analyzed by Image-Pro Plus 5.0 software. (b) Effect of p53 on OLA-induced MDA levels and SOD and CAT activities in HCT116 cells. (c) The impact of p53 on biomarkers of the Nrf2/HO-1 pathway in HCT116 cells. (d) Interference of p53 by plasmid on OLA-induced MDA levels and SOD and CAT activities in L02 cells. (e) Interference of p53 by plasmid on biomarkers of the Nrf2/HO-1 pathway in L02 cells. The results represent the means ± SD of three independent experiments. ^∗^*p* < 0.05 and ^∗∗^*p* < 0.01, compared with the control; ^#^*p* < 0.05 and ^##^*p* < 0.01, compared with the p53 normal group.

Thus, we investigated the effect of p53 on intracellular ROS levels induced by OLA. The results showed that ROS production was significantly reduced in the HCT116 (p53-/-) group (decreased from 4.5- to 2.6-folds treated with 800 *μ*g/mL OLA), compared with the p53+/+ group (Figure 6(a)). Meanwhile, the p53 knockout reduced OLA-induced oxidative stress and inhibited antioxidant pathway Nrf2/HO-1 activation in HCT116 cells (Figures 6(b) and 6(c)). Furthermore, we used the L02 cell model to examine the role of p53 on oxidative stress and the Nrf2/HO-1 pathway. The results showed that knockdown of p53 further reduced OLA-induced oxidative stress and considerably enhanced the activities of SOD and CAT, compared to the OLA-alone group (Figure 6(d)). As shown in Figure 6(e), knockdown of p53 inhibited the activation of the Nrf2/HO-1 pathway treated with OLA, which is consistent with the result of the HCT116 cells.

### 3.7. Ablation of p53 Attenuated OLA-Induced Mitochondrial Dysfunction In Vitro

**Figure 7:**
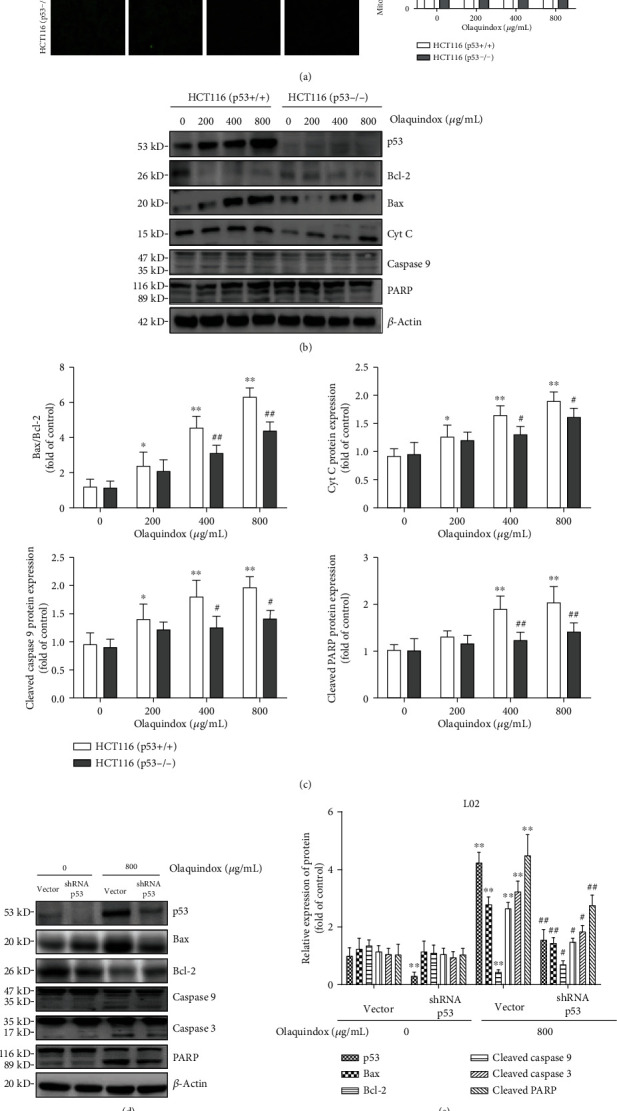
Ablation of p53 attenuated OLA-induced mitochondrial dysfunction in vitro. (a) Cells were stained by Rhodamine 123 (10 *μ*g/mL) and observed under a fluorescence microscope (400x). (b) Expressions of p53 Bax, Bcl-2, Cyt C caspase 9, and PARP in HCT116 cells were detected by western blotting analysis. (c) The densitometry analysis of p53, Bax/Bcl-2, Cyt C, caspase 9, and PARP by the ImageJ 1.46 software. (d) Expressions of p53 Bax, Bcl-2, caspase 9, caspase 3, and PARP in L02 cells were detected by western blotting analysis. (e) The densitometry analysis via the ImageJ 1.46 software. The results represent the means ± SD of three independent experiments. ^∗^*p* < 0.05 and ^∗∗^*p* < 0.01, compared with the control; ^#^*p* < 0.05 and ^##^*p* < 0.01, compared with the p53 normal groups.

As shown in Figure 7(a), compared with the HCT116 (p53+/+) group, the mitochondrial membrane potential of the HCT116 (p53-/-) group was restored after treatment with OLA, and the 800 *μ*g/mL group was increased from 38.3% to 51.6%. Then, we examined the marker protein of the mitochondrial apoptosis pathway. Compared with the HCT116 (p53+/+) group, the ratio of Bax/Bcl-2 was reduced, and the release of Cyt C from the mitochondria into the cytoplasm was also reduced in the HCT116 (p53-/-) group (Figures 7(b) and 7(c)). The deletion of p53 significantly inhibited the cleaving of caspase 9 and PARP protein expression induced by OLA 400 and 800 *μ*g/mL (Figures 5(b) and 5(c)). As for L02 cells, the p53 interfering plasmid significantly inhibited the protein expression of p53 (Figure 7(d)). Furthermore, interfering with p53 observably inhibited the OLA-induced mitochondrial apoptosis pathway (Figures 7(d) and 7(e)).

### 3.8. Loss of p53 Partially Inhibited the JNK/P38 Pathway and Activated the Autophagy Pathway

**Figure 8:**
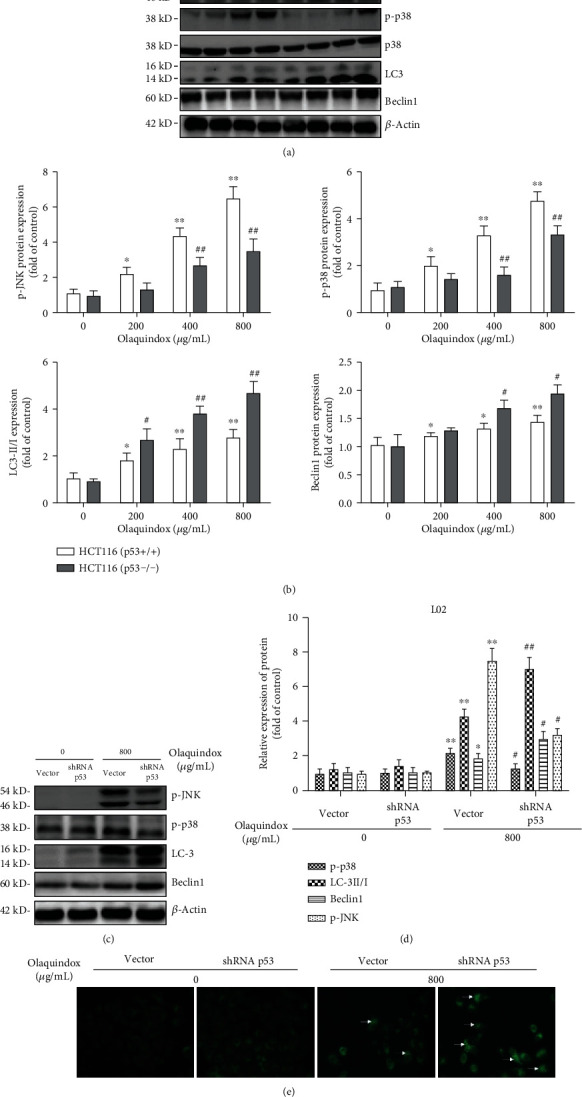
Loss of p53 partially inhibited JNK/p38 pathway and activated autophagy pathway. (a) Western blot analysis for JNK, p38, LC3, and Beclin1 in HCT116 cells. (b) The densitometric analysis of the bands in HCT116 cells were performed via the ImageJ 1.46 software. (c) Western blot analysis for JNK, p38, LC3, and Beclin1 in L02 cells. (d) The densitometric analyses of the bands in L02 cells were performed by the ImageJ 1.46 software. (e) Fluorescence microscopy was used to observe the distribution of autophagosomes in L02 cells after MDC staining (400x). The results represent the means ± SD of three independent experiments. ^∗^*p* < 0.05 and ^∗∗^*p* < 0.01, compared with the control; ^#^*p* < 0.05 and ^##^*p* < 0.01, compared with p53 normal groups.

As shown in Figure 8(a), the expression levels of p-JNK and p-p38 in the OLA 400 and 800 *μ*g/mL groups were significantly reduced after p53 deletion, compared to those of the HCT116 (p53+/+) groups. Furthermore, the expression of LC3 and Beclin1 induced by OLA increased significantly in the p53 knockout group (Figure 8(a)), indicating that p53 may have inhibited the activation of the autophagy pathway. In addition, interfering with p53 inhibited the OLA-induced activation of JNK/p38 pathways and activated the expression of LC3 and Beclin1 in L02 cells (Figure 8(c)). Moreover, fluorescence microscopy was performed with MDC staining to qualitatively analyze the occurrence of autophagy in L02 cells. As shown in Figure 8(e), the fluorescence intensity was weak, and the punctate structure was less in the vector and shRNA groups without OLA. After the interference with p53, the fluorescence intensity was significantly higher than that of the normal p53 group treated with OLA, and obvious aggregation of fluorescent particles could be seen in the cytoplasm, indicating that interference with p53 could cause autophagy in L02 cells (Figure 8(e)).

## 4. Discussion

The p53 signaling pathway participated in the transmission of apoptotic signals in the process of cell death [24]. It suggested that the balance between oxidative stress and antioxidant systems was critical for cell survival in the process of p53-induced oxidative stress and apoptosis [25]. The effects of p53 on antioxidation or prooxidation depend on the level of oxidative stress production. Although OLA has been banned as a feed additive in many countries due to the reproductive toxicity and genotoxicity [3], the abuse of OLA in feed additives of food-production animals is still serious, as well as other quinoxalines, such as quinocetone and mequindox. The liver may be the main target organ for toxicity of OLA with the evidence that high concentrations of OLA could noticeably induce liver injury and liver dysfunction [26, 27].

To investigate the role of p53 in OLA-induced liver damage, a mouse model and a cell model treated by OLA were established. It has been known that AST and ALT mainly exist in the liver. Under oxidative stress, damaged liver cell membranes lead to the release of AST and ALT into the blood, which significantly increases the content of AST and ALT in the serum. In the current study, the data showed that the serum levels of ALT and AST were coincident with the histopathological and ultrastructural damage of mice treated with OLA in a dose-dependent manner (Figure 1), indicating that the mouse model was successfully established. Oxidative stress is the imbalance between oxidation and antioxidation in the body, which is recognized as an important factor in aging and disease [28]. MDA is a biomarker of lipid peroxidation, and SOD and CAT belong to the enzyme antioxidant system in vivo [29]. SOD is the first enzyme in the antioxidant enzyme system to combine with the active oxygen radical in vivo, which could degrade the superoxide anion (O^2-^) to form H_2_O_2_ and then combine CAT with H_2_O_2_ to decompose it into water and oxygen molecules [13]. In this study, MDA, SOD, and CAT in the liver of mice were further detected. The results revealed that the level of MDA was remarkably enhanced, whereas SOD and CAT activities declined after continuous administration of OLA for 28 days (Figures 2(d)–2(f)), indicating that oxidative stress was involved in OLA-induced liver injury. Previous studies showed that the intracellular ROS content was significantly increased after treatment with OLA, which was consistent with the results of this study (Figure 6(a)). Thus, the production of ROS was considered as an early factor of cytotoxicity induced by OLA [1].

Moreover, our consequences displayed that the p53 pathway was dramatically activated in mouse and cell models (Figure 1(g) and Figures 7(b) and 7(d)), indicating that hepatotoxicity induced by OLA was associated with the p53 pathway. Therefore, p53 knockout mice and p53 deletion cell lines were used to further investigate OLA-induced liver damage and the underlying molecular mechanisms. Our results showed that p53 knockout alleviated OLA-induced liver injury and oxidative stress (Figure 3). Accumulating evidences showed that knockout of p53 alleviated the damage caused by oxidative stress [30, 31]. Previous reports showed that depletion of p53 attenuated cocaine-induced hepatotoxicity in mice, which demonstrated that the inhibition of p53 was important for protection against oxidative stress and proapoptotic events [32]. Moreover, p53 deletion prevented ischemic renal injury through attenuating both renal histologic damage and oxidative stress [31]. Importantly, in response to high levels of oxidative stress, p53 shows prooxidative activity, which further increases the stress level and leads to cell apoptosis [33]. Our further results discovered that p53 deletion increased OLA-induced cell viability (Figures 5(a) and 5(e)), while decreasing OLA-induced apoptosis (Figures 5(d) and 5(f)) and production of ROS (Figure 6(a)), indicating that it may be related to the reduction of OLA-induced oxidative stress by the absence of p53. Previous research has shown that apoptosis was completely abrogated by ionizing radiation in the absence of p53 [34]. p53 exerts as an antioxidant, neutralizing free radicals and protecting the genome from ROS-induced injury, which regulates many antioxidant genes and protects cells from low levels of ROS-induced damage [35]. However, high contents of ROS or sustaining stress will increase p53 and stimulate the oxidant to further promote ROS generation. Therefore, triggering the p53-dependent apoptosis could contribute to clear the injured cells [36, 37]. Furthermore, p53 also affects the production of mitochondrial ROS and tilts the redox balance toward cell death [38]. The above results indicated that OLA-induced liver damage involved the crosstalk of oxidative stress and p53.

Not surprisingly, previous reports showed that the mitochondrial apoptosis pathway was the main pathway of OLA-induced apoptosis [5, 39]. Mitochondrial damage increases the permeability of the mitochondrial membrane, leading to the release of Cyt C into the cytoplasm, which in turn results in a caspase cascade reaction, thus leading to apoptosis [23]. Furthermore, p53 could activate Bax directly, which leads to the decrease of MMP, followed with the release of Cyt C and activation of caspases, which triggers apoptosis [40]. In our study, p53 knockout attenuated OLA-induced mitochondrial damage and the levels of caspase 3 and caspase 9 in mice (Figure 4). Our further results discovered that knockout of p53 significantly alleviated the decrease of the mitochondrial membrane potential (Figure 7(a)) and inhibited the OLA-induced mitochondrial apoptosis pathway in HCT116 and L02 cells (Figures 7(b) and 7(d)). Taken together, these investigations implied that p53 was able to dependently regulate OLA-induced mitochondrial dysfunction and apoptosis in vivo and in vitro. Cell apoptosis, an internal response to definite pressures, happens via p53-dependent and p53-independent pathways [41]. Notably, certain stressors, such as anoxia, oxidative damage, drug treatment, and oncogene abnormalities, could eventually trigger the p53-mediated cell death pathway in the cell [42]. Based on the above results, we further explored the mechanism of p53-regulated OLA-induced liver damage in vivo and in vitro.

Our findings showed that knockout or knockdown of p53 effectively alleviated the activation of the JNK/p38 pathway (Figures 8(a) and 8(c)), suggesting that p53 may act as the upstream of JNK/p38 pathways in OLA-induced toxicity. Our previous study demonstrated that JNK/p38 inhibitors (SP600125 and SB203580) significantly affected OLA-induced apoptosis [17], indicating that the JNK/P38 pathway participated in OLA-induced apoptosis. The JNK/p38 signaling pathway is activated or altered in a variety of cancers and regulates a variety of cellular processes including survival, proliferation, metabolism, angiogenesis, and metastasis [43]. p53 activation has been shown to induce more autophagosomes in wild-type p53 MEF cells than in p53-inactive MEF cells in response to stress, which occurs through p53 inhibition of the AKT/mTOR pathway or through regulation of certain p53 target genes directly involved in autophagy [44]. More intriguingly, LC3 and Beclin1 were further activated in OLA treatment after p53 knockout or knockdown, followed with obvious aggregation of autophagosomes in the cytoplasm (Figure 8(e)), suggesting that inhibition of p53 enhanced autophagy in L02 cells. It has been demonstrated that p53 knockout in HCT116 cells could increase the expression of cytosol HMGB1 and induce autophagy [45]. In our previous study, inhibition of autophagy significantly increased the OLA-induced apoptotic rate [14], suggesting that the interaction between apoptosis and autophagy was mutual inhibition. Collectively, our results provided a support that p53 knockout alleviated OLA-induced liver injury, which may be associated with upregulation of the autophagy pathway and inhibition of the JNK/p38 pathway.

## 5. Conclusions

**Figure 9:**
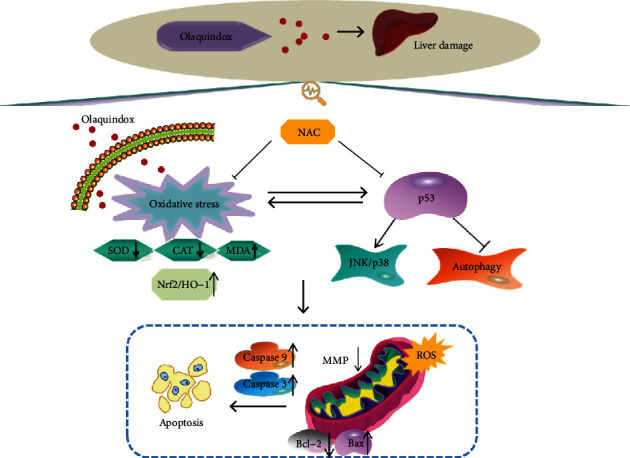
A proposed signaling pathway of p53 on OLA-induced liver damage and oxidative stress in vivo and in vitro.

In conclusion, as illustrated in Figure 9, our findings demonstrated that p53 primarily played a proapoptosis role in OLA-induced liver damage. Knockout of p53 effectively protected liver injury against oxidative stress and ROS production, which was largely dependent on inhibition of the JNK/p38 signaling pathway and upregulation of the autophagy via activating LC3 and Beclin1. Therefore, the mechanism of p53 in response to oxidative stress and the outcomes of the interaction between p53 and other signaling pathways need further elucidation. This study will shed light on the molecular toxicity of OLA and other quinoxalines.

## Data Availability

All relevant data in the current study are available from the corresponding author (tssfj@cau.edu.cn) on request.

## References

[1] Chen Q., Tang S., Jin X. (2009). Investigation of the genotoxicity of quinocetone, carbadox and olaquindox in vitro using Vero cells. *Food and Chemical Toxicology*.

[2] Wang X., Martinez M. A., Cheng G. (2016). The critical role of oxidative stress in the toxicity and metabolism of quinoxaline 1,4-di-N-oxides in vitro and in vivo. *Drug Metabolism Reviews*.

[3] Ihsan A., Wang X., Zhang W. (2013). Genotoxicity of quinocetone, cyadox and olaquindox in vitro and in vivo. *Food and Chemical Toxicology*.

[4] Zou J., Chen Q., Tang S. (2009). Olaquindox-induced genotoxicity and oxidative DNA damage in human hepatoma G2 (HepG2) cells. *Mutation Research*.

[5] Li D., Dai C., Zhou Y. (2016). Effect of GADD45a on olaquindox-induced apoptosis in human hepatoma G2 cells: involvement of mitochondrial dysfunction. *Environmental Toxicology and Pharmacology*.

[6] Zhao W. X., Tang S. S., Jin X. (2013). Olaquindox-induced apoptosis is suppressed through p38 MAPK and ROS-mediated JNK pathways in HepG2 cells. *Cell Biology and Toxicology*.

[7] Dai C., Li B., Zhou Y. (2016). Curcumin attenuates quinocetone induced apoptosis and inflammation via the opposite modulation of Nrf2/HO-1 and NF-kB pathway in human hepatocyte L02 cells. *Food and Chemical Toxicology*.

[8] Dai C., Tang S., Li D., Zhao K., Xiao X. (2015). Curcumin attenuates quinocetone-induced oxidative stress and genotoxicity in human hepatocyte L02 cells. *Toxicology Mechanisms and Methods*.

[9] Haykal J., Geara F., Haddadin M. J., Smith C. A., Gali-Muhtasib H. (2009). The radiosensitizer 2-benzoyl-3-phenyl-6,7-dichloroquinoxaline 1,4-dioxide induces DNA damage in EMT-6 mammary carcinoma cells. *Radiation Oncology*.

[10] Liu Q., Lei Z., Gu C. (2019). Mequindox induces apoptosis, DNA damage, and carcinogenicity in Wistar rats. *Food and Chemical Toxicology*.

[11] Huang X. J., Wang X., Ihsan A. (2010). Interactions of NADPH oxidase, renin-angiotensin-aldosterone system and reactive oxygen species in mequindox-mediated aldosterone secretion in Wistar rats. *Toxicology Letters*.

[12] Ihsan A., Wang X., Huang X. J. (2010). Acute and subchronic toxicological evaluation of mequindox in Wistar rats. *Regulatory Toxicology and Pharmacology*.

[13] Dai C., Li D., Gong L., Xiao X., Tang S. (2016). Curcumin ameliorates furazolidone-induced DNA damage and apoptosis in human hepatocyte L02 cells by inhibiting ROS production and mitochondrial pathway. *Molecules*.

[14] Zhao D., Wang C., Tang S. (2015). Reactive oxygen species-dependent JNK downregulated olaquindox-induced autophagy in HepG2 cells. *Journal of Applied Toxicology*.

[15] Li D., Dai C., Yang X., Li B., Xiao X., Tang S. (2017). GADD45a regulates olaquindox-induced DNA damage and S-phase arrest in human hepatoma G2 cells via JNK/p38 pathways. *Molecules*.

[16] Li D., Dai C., Yang X. (2017). Critical role of p21 on olaquindox-induced mitochondrial apoptosis and S-phase arrest involves activation of PI3K/AKT and inhibition of Nrf2/HO-1pathway. *Food and Chemical Toxicology*.

[17] Li D., Zhao K., Yang X., Xiao X., Tang S. (2017). TCS2 increases olaquindox-induced apoptosis by upregulation of ROS production and downregulation of autophagy in HEK293 cells. *Molecules*.

[18] Polyak K., Xia Y., Zweier J. L., Kinzler K. W., Vogelstein B. (1997). A model for p53-induced apoptosis. *Nature*.

[19] Zhang C., Wang C., Tang S. (2013). TNFR1/TNF-*α* and mitochondria interrelated signaling pathway mediates quinocetone-induced apoptosis in HepG2 cells. *Food and Chemical Toxicology*.

[20] El-Khatib M., Geara F., Haddadin M. J., Gali-Muhtasib H. (2010). Cell death by the quinoxaline dioxide DCQ in human colon cancer cells is enhanced under hypoxia and is independent of p53 and p21. *Radiation Oncology*.

[21] Yang Y., Jiang L., She Y. (2015). Olaquindox induces DNA damage via the lysosomal and mitochondrial pathway involving ROS production and p53 activation in HEK293 cells. *Environmental Toxicology and Pharmacology*.

[22] Dai C., Li J., Tang S., Li J., Xiao X. (2014). Colistin-induced nephrotoxicity in mice involves the mitochondrial, death receptor, and endoplasmic reticulum pathways. *Antimicrobial Agents and Chemotherapy*.

[23] Dai C., Xiao X., Li D. (2018). Chloroquine ameliorates carbon tetrachloride-induced acute liver injury in mice via the concomitant inhibition of inflammation and induction of apoptosis. *Cell Death & Disease*.

[24] Muller P. A., Vousden K. H. (2014). Mutant p53 in cancer: new functions and therapeutic opportunities. *Cancer Cell*.

[25] Hussain S. P., Amstad P., He P. (2004). p53-induced up-regulation of MnSOD and GPx but not catalase increases oxidative stress and apoptosis. *Cancer Research*.

[26] Cheng G., Sa W., Cao C. (2016). Quinoxaline 1,4-di-N-oxides: biological activities and mechanisms of actions. *Frontiers in Pharmacology*.

[27] Zhou J., Li C., Wang L., Ji H., Zhu T. (2015). Hepatoprotective effects of a Chinese herbal formulation, Yingchen decoction, on olaquindox-induced hepatopancreas injury in Jian carp (Cyprinus carpio var. Jian). *Fish Physiology and Biochemistry*.

[28] Sies H. (2015). Oxidative stress: a concept in redox biology and medicine. *Redox Biology*.

[29] Victor V. M., Rocha M., Esplugues J. V., De la Fuente M. (2005). Role of free radicals in sepsis: antioxidant therapy. *Current Pharmaceutical Design*.

[30] Yi J. Y., Hirabayashi Y., Choi Y. K. (2009). Benzene activates caspase-4 and -12 at the transcription level, without an association with apoptosis, in mouse bone marrow cells lacking the p53 gene. *Archives of Toxicology*.

[31] Ying Y., Kim J., Westphal S. N., Long K. E., Padanilam B. J. (2014). Targeted deletion of p53 in the proximal tubule prevents ischemic renal injury. *Journal of the American Society of Nephrology*.

[32] Mai H. N., Sharma G., Sharma N. (2019). Genetic depletion of p53 attenuates cocaine-induced hepatotoxicity in mice. *Biochimie*.

[33] Liu D., Xu Y. (2011). p53, oxidative stress, and aging. *Antioxidants & Redox Signaling*.

[34] Li T., Kon N., Jiang L. (2012). Tumor suppression in the absence of p53-mediated cell-cycle arrest, apoptosis, and senescence. *Cell*.

[35] Sablina A. A., Budanov A. V., Ilyinskaya G. V., Agapova L. S., Kravchenko J. E., Chumakov P. M. (2005). The antioxidant function of the p53 tumor suppressor. *Nature Medicine*.

[36] Bensaad K., Vousden K. H. (2005). Savior and slayer: the two faces of p53. *Nature Medicine*.

[37] Chen W., Jiang T., Wang H. (2012). Does Nrf2 contribute to p53-mediated control of cell survival and death?. *Antioxidants & Redox Signaling*.

[38] Wang D. B., Kinoshita C., Kinoshita Y., Morrison R. S. (2014). p53 and mitochondrial function in neurons. *Biochimica et Biophysica Acta*.

[39] Zou J., Chen Q., Jin X. (2011). Olaquindox induces apoptosis through the mitochondrial pathway in HepG2 cells. *Toxicology*.

[40] Kelly K. J., Sandoval R. M., Dunn K. W., Molitoris B. A., Dagher P. C. (2003). A novel method to determine specificity and sensitivity of the TUNEL reaction in the quantitation of apoptosis. *American Journal of Physiology. Cell Physiology*.

[41] Fridman J. S., Lowe S. W. (2003). Control of apoptosis by p53. *Oncogene*.

[42] Ahn B. Y., Trinh D. L., Zajchowski L. D., Lee B., Elwi A. N., Kim S. W. (2010). Tid1 is a new regulator of p53 mitochondrial translocation and apoptosis in cancer. *Oncogene*.

[43] Ersahin T., Tuncbag N., Cetin-Atalay R. (2015). The PI3K/AKT/mTOR interactive pathway. *Molecular BioSystems*.

[44] Feng Z., Zhang H., Levine A. J., Jin S. (2005). The coordinate regulation of the p53 and mTOR pathways in cells. *Proceedings of the National Academy of Sciences of the United States of America*.

[45] Livesey K. M., Kang R., Vernon P. (2012). p53/HMGB1 complexes regulate autophagy and apoptosis. *Cancer Research*.

